# The Validity of Sensors and Model in the Lane Change Control Process †

**DOI:** 10.3390/s23104738

**Published:** 2023-05-14

**Authors:** Andrzej Dębowski, Jakub Jan Faryński, Dariusz Piotr Żardecki

**Affiliations:** 1 Faculty of Mechanical Engineering, Military University of Technology, Gen. Sylwestra Kaliskiego 2, 00-908 Warsaw, Poland; andrzej.debowski@wat.edu.pl (A.D.); dariusz.zardecki@wat.edu.pl (D.P.Ż.); 2 Doctoral School, Military University of Technology, Gen. Sylwestra Kaliskiego 2, 00-908 Warsaw, Poland

**Keywords:** bicycle model, four-wheel steering vehicle, lane change manoeuvre, “bang-bang” signal, sensors, sensitivity, signal disturbances, autonomous vehicle, Matlab and Simulink

## Abstract

The paper demonstrates the validity of sensors and the model in the algorithm for a lane change controller. The paper presents the systematic derivation of the chosen model from the ground up and the important role played by the sensors used in this system. The whole concept of the system on which the tests were carried out is presented step by step. Simulations were realised in the Matlab and Simulink environments. Preliminary tests were performed to confirm the need for the controller in a closed-loop system. On the other hand, sensitivity (the influence of noise and offset) studies showed the advantages and disadvantages of the developed algorithm. This allowed us to create a research path for future work with the aim of improving the operation of the proposed system.

## 1. Introduction

It was decided to take up the above topic of the article, first of all, due to the authors’ work on the topic of automatic control for the lane change manoeuvre by a car with four steering wheels (4WS). Secondly, an important and large part of it concerns the sensitivity of the control system to the presence of noise and offset in the measurement signals sent from the sensors to the controller, which fits in with the theme of the magazine.

It was decided to work on the 4WS system because it is one of the elements supporting the active safety of the car. The idea of its use fits well with its application in an autonomous vehicle—increasing manoeuvrability at high speeds (e.g., in a parking lot) and increased stability when driving at higher speeds (e.g., on a highway) [1].

The concept of an autonomous vehicle is becoming increasingly common. The development of vehicles in this respect is linked to the increasing automation of the individual processes they perform. In the case of the topic addressed by the authors, this is automatic navigation [2,3] and automatic obstacle avoidance [4,5]. The lane change manoeuvre is encountered in situations of avoiding, overtaking, and when an unexpected obstacle suddenly appears on the road. It can therefore be said to be one of the basic manoeuvres from which other, even more complex manoeuvres are performed. Therefore, when studying the steering algorithm, it is reflected in lane-change studies. This topic is addressed in many scientific papers [4,5,6,7,8,9,10,11,12]. Researchers are mostly concerned with the preparatory phase. This is a very important issue related to the driver’s (controller’s) decision [6]. This applies to the situation of choice, when a pre-selection situation arises and one has to take into account what is happening in the adjacent lane [7,8]. The question then arises as to whether it is empty and whether the manoeuvre can be executed [12]. Assuming that a vehicle is coming from the opposite direction, the driver is faced with a difficult choice [9]. The driver has to decide whether to hit the obstacle after braking or whether to attempt to change lanes, hoping to do so in time before being hit by the vehicle on the opposite side [11]. Lane change manoeuvre environment detection by the driver (autonomous vehicle) concerns the preparatory phase of a lane change manoeuvre [10]. The second phase of this manoeuvre is the execution phase, i.e., the actual control process, when information is already provided to the system regarding the possibility of executing the manoeuvre. In this phase, cooperation between the controller, the measuring system (the vehicle movement measurement system), and the vehicle is crucial [4]. Information must be provided on an ongoing basis, as the controller must obtain information on how the vehicle should move. The main goal is to formulate a controller that will make the displacement of the vehicle appropriate given the dynamics of the movement, the geometry of the vehicle, and the obstacle to be avoided [4,5]. The execution phase is crucial for the active safety of the steering car. 

Among other things, the sensors in the vehicle’s motion measurement system and the environmental (road) detection system play a key role in this issue, as they provide the necessary information to the controller, which translates into the operation of the entire system [13]. It is also worth mentioning at this point that sensors also introduce various types of imperfections into the whole system, such as measurement noise or offsets, among others. This issue has been addressed in several papers [14,15,16]. It is important that the developed control algorithm, in spite of the existing imperfections, is able to keep the vehicle in the assumed trajectory and perform the requested manoeuvre. The issue of the sensitivity of automatic control to the occurrence of measurement disturbances seems to be particularly important for evaluating the performance of our proposed lane change controller, which is also an important element of this article.

In determining lane change control algorithms, a mathematical model that describes the dynamics of vehicle movement in the plane of the road plays a key role. Such a model is the so-called “bicycle model,” which is applied to both two-wheel steerable (2WS) and four-wheel steerable (4WS) cars. In papers [17,18], the bicycle model is used together with the MPC (Model Predictive Control) technique for the process of lane change. Some review works related to the bicycle model can also be found [19,20]. Among other things, it has also found application in various computer simulations [21], real-world studies [22], the study of variable vehicle dynamics [23], vehicle performance [24], active steering [25,26], the comparison of 2WS and 4WS vehicle control [27], and control system design [28]. 

Many papers show that the bicycle model is a very important element in lateral vehicle dynamics issues, while the derivation of the model from the ground up is rare. Therefore, this paper also presents its derivation. This allows for a better understanding of the structure of the model and shows its validity in relation to its dependence on individual parameters. The transformation of the bicycle model from the form of differential equations to the transmittance form is also presented in this paper. The transmittance form of the bicycle model is very helpful in the synthesis of control algorithms. 

The lateral dynamics model of the 4WS car in the form of differential equations is a classic model used in the works of many authors. An important novelty is the transformation of equations into the transfer function form, the reduction of the transfer function model, and, on this basis, the synthesis of the controller. This is the authors’ original contribution to the development of 4WS vehicle control technology.

## 2. Concept of the Steering System

The proposed control system consists of a reference signal generator and regulators [19]. The reference signal generator is based on the lane change process reference model. It generates a reference steering signal and corresponding reference output signals. According to the theory of control systems and the practise of drivers, the reference steering signal has a “bang-bang” type signal. This signal is corrected on the basis of the correction signals from the regulators, which it goes to the summing node with. The regulators correct the reference control signal so that, after correction, the actual control signal acting on the car ensures the minimization of errors, and finally, a good implementation of the lane change process. The algorithms of the regulators are also based on a reference model describing the lane change process. The reference model is therefore crucial for the design of the lane change controller. As shown in the literature analyses [4,5,19,29,30,31,32], the bicycle model turned out to be the correct reference model used in many solutions by other authors.

**Figure 1:**
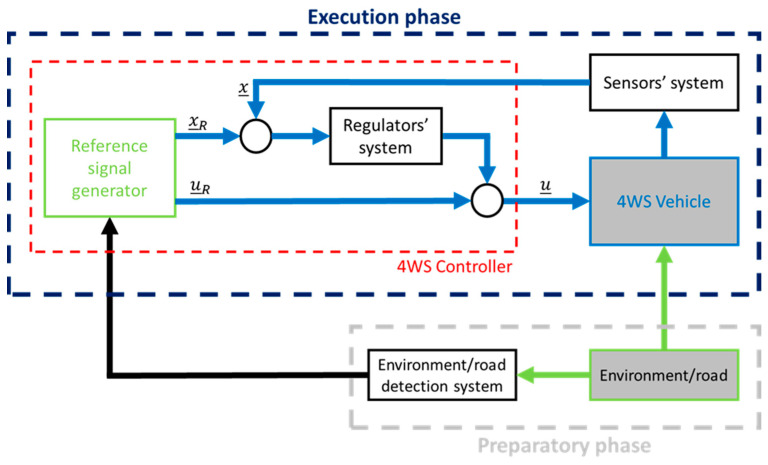
The idea of the 4WS control system. Where: $\underline{x}$ —output signals, $\underline{u}$ —steering signals, ${\underline{x}}_{R}$ —reference output signals, ${\underline{u}}_{R}$ —reference steering signal.

An example of a control method for an autonomous vehicle is shown in the control diagram below (Figure 1).

## 3. Derivation of the Model

**Figure 2:**
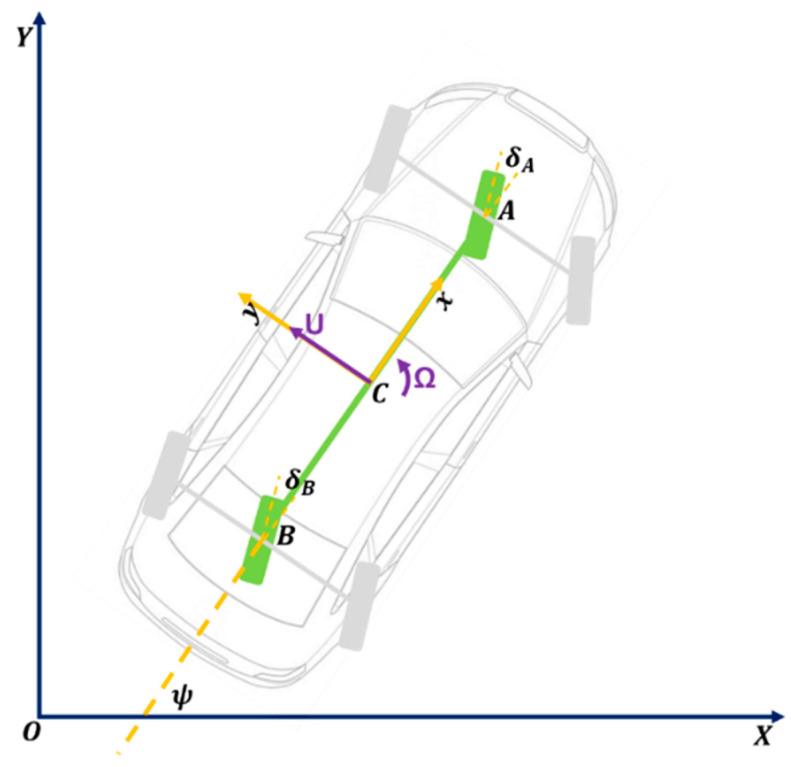
Idea of a bicycle model.

The bicycle model is a plane model with three degrees of freedom. It assumes that the vehicle’s centre of mass is located on the plane of the road. The vehicle itself is symmetrical with respect to the longitudinal vertical plane. The axles are reduced to individual wheels. This model assumes that the left and right wheels of the vehicle generate the same lateral forces and depend only on the drift angle of the entire vehicle axis (Figure 2) [33].

This means that the description of a car is equivalent to that of a single-track vehicle. An important simplification here is the omission of vehicle rollovers. By using this model, predictions of vehicle behaviour can be calculated in real-time and form the basis for the calculation of corrective signals for car stabilisation. The degrees of freedom taken into account by the model are two translational coordinates of the centre of mass (*X*, *Y*) and one rotational—yaw. This model uses a linear tyre model. It is one of the simpler mathematical models that assumes that the forces acting under the wheel are as follows:

(1) $${F_{A,B}}_{\left(x\right)}=C_{A,B}\cdot \lambda_{A,B}$$

(2) $${F_{A,B}}_{\left(y\right)}=K_{A,B}\cdot \alpha_{A,B}$$

Forces ${F_{A,B}}_{\left(x\right)}$ and ${F_{A,B}}_{\left(y\right)}$ are proportional to the longitudinal wheel slip ( $\lambda_{A,B}$ ) and its drift angle ( $\alpha_{A,B}$ ). Slip resistance coefficients ( $C_{A,B}$ ) and tyre cornering stiffness ( $K_{A,B}$ ) are constant values. An example of methodology for determining drift resistance factors can be found in [34]. Such a model is accurate for wheel slip $\lambda_{A,B}<0.15$ and the wheel drift angle $\alpha_{A,B}<0.1$ rad. For larger values of wheel slip or tyre cornering stiffness, the forces calculated from the real model are higher than the real forces acting at the contact between the tyre and the road [35].

**Figure 3:**
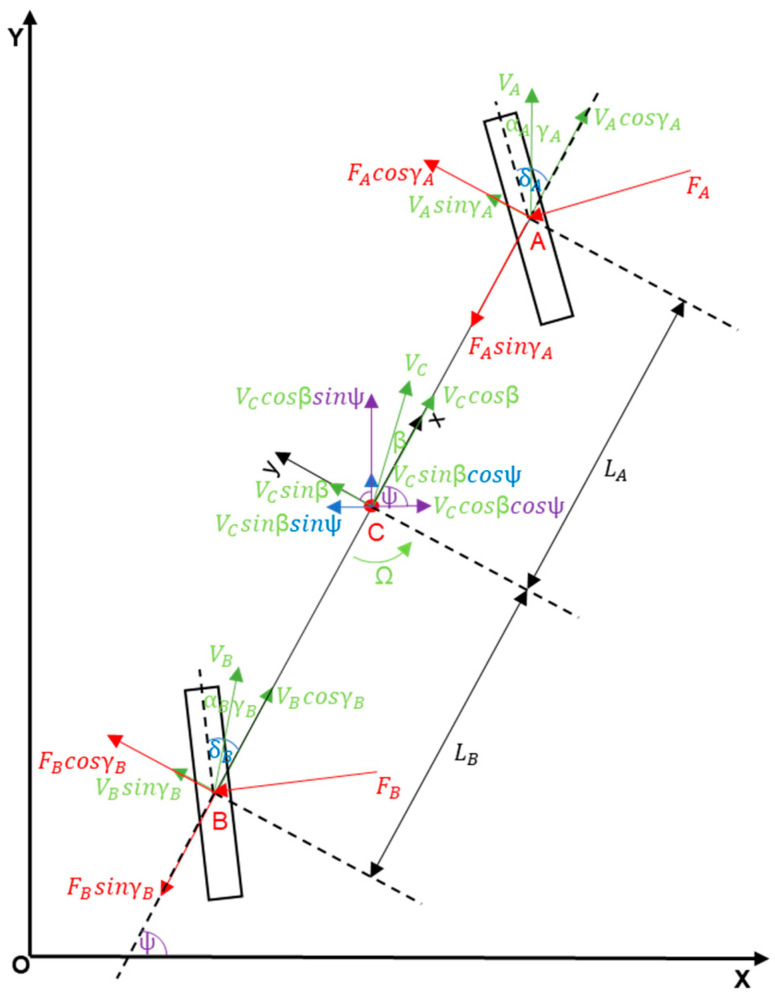
Bicycle model, including all forces and moments.

At the very beginning, it is necessary to consider all the forces and moments that will be needed to create the details of the model in Figure 3, which are as follows:

-
(X,Y) —global coordinate system,
-
(x,y) —local coordinate system,
-
m —vehicle mass,
-
J —mass moment of inertia ( $J_{zz}$ to p. C),
-
$L_{A},L_{B}$ —distances AC and BC between the centre of mass to the front/rear axes,
-
$K_{A},K_{B}$ —front and rear tyre cornering stiffness (yaw coefficients to p. A and B),
-
$\delta_{A},\delta_{B}$ —front and rear steering angles,
-
Ω —angular velocity of the vehicle,
-
V —longitudinal vehicle speed in the local coordinate system,
-
U —lateral vehicle speed in the local coordinate system,
-
$V_{C}$ —velocity in the centre of mass,
-
$a_{C}$ —acceleration in the centre of mass,
-
β —drift angle of the vehicle; $\beta =arctg\left(\frac{V_{C}\sin\beta }{V_{C}\cos\beta }\right)$ ,
-
$\alpha_{A,B}$ —drift angles (to p. A and B),
-
$\gamma_{A,B}$ —subtraction of the steering angles and drift angles (to p. A and B).

Another important element is the adoption of assumptions and simplifications in the model under consideration. Otherwise, the individual expressions of the model would take on a very complicated structure, which would make the analysis of such a model very difficult at a later stage. 

Assumptions:

The considered issues concern the control of motion of a 4WS vehicle travelling at a steady speed on a straight, level road with good adhesion, so that the well-known “bicycle model” can be used to describe the dynamics:

- The vehicle is treated as a moving single-mass planar object on sprung, twisting pneumatics, symmetrical about its longitudinal axis.
- The road reaction forces are perpendicular to the wheel rim side planes and depend directly (functional dependence) on the drift angles, so the angles between the wheel rim side planes and the wheel tyre line marks.
- The road reaction forces are related to the points defined by the vertical projections of the wheel axis centres.

The adopted simplifications:

-
$V_{C}\cos\beta \approx V_{A}=V_{B}=V=constant$ ,
-
$V_{C}\sin\beta \approx U$ ,
-
$\sin\gamma_{A}\approx \gamma_{A}$ ,
-
$\cos\gamma_{A}\approx 1$ ,
-
$\sin\gamma_{B}\approx \gamma_{B}$ ,
-
$\cos\gamma_{B}\approx 1$ ,
-
$\tan\gamma_{A}\approx \gamma_{A}$ ,
-
$\tan\gamma_{B}\approx \gamma_{B}$ .

With the model scheme and its simplifications thus prepared, one can proceed with the calculations. The global and local coordinate systems should be analysed separately, given that their respective properties are interdependent and will need to be exploited. The calculations will start with the local coordinate system and be divided into several steps.

Calculations against (x,y) :

(1) Forces calculations:

(3) $$\sum F_{y}=F_{A}+F_{B}\;(\mathrm{from}\;\mathrm{simplifications}\;\mathrm{adopted})$$

(4) $$F_{A}+F_{B}=ma_{Cy}$$

(5) $$V_{C}=V+U$$

(6) $$a_{Cy}=\dot{U}+V\Omega$$

By substituting the Relation (6) into Equation (4), one obtains:

(7) $$F_{A}+F_{B}=m(\dot{U}+V\Omega )$$

Once the above calculations are made, we are able to proceed to the next step.

(2) Moments of force calculations:

(8) $$\sum M=J\dot{\Omega }$$

(9) $$\sum M_{y}=F_{A}L_{A}-F_{B}L_{B}\;(\mathrm{from}\;\mathrm{simplifications}\;\mathrm{adopted})$$

(10) $$F_{A}L_{A}-F_{B}L_{B}=J\dot{\Omega }$$

The next step will be calculating the tyre model. As mentioned earlier, the bicycle model uses a linear tyre model.

(3) Tyre model calculations:

Front tyre:

The lateral speed of the front tyre is the sum of the lateral speed at the centre of mass and the angular speed at the front.

(11) $$V_{A}\gamma_{A}=U+\Omega L_{A}\;(\mathrm{from}\;\mathrm{simplifications}\;\mathrm{adopted})$$

(12) $$\alpha_{A}+\gamma_{A}=\delta_{A}$$

(13) $$\alpha_{A}=\delta_{A}-\frac{U+\Omega L_{A}}{V_{A}}$$

(14) $$\mathrm{from}\;(2):\;F_{A}=K_{A}\alpha_{A}$$

(15) $$F_{A}=K_{A}(\delta_{A}-\frac{U+\Omega L_{A}}{V_{A}})$$

Rear tyre:

The lateral speed of the rear tyre is the result of the subtraction of the lateral speed at the centre of mass and the angular speed at the rear.

(16) $$V_{B}\gamma_{B}=U-\Omega L_{B}\;(\mathrm{from}\;\mathrm{simplifications}\;\mathrm{adopted})$$

(17) $$\alpha_{B}+\gamma_{B}=\delta_{B}$$

(18) $$\alpha_{B}=\delta_{B}-\frac{U-\Omega L_{B}}{V_{B}}$$

(19) $$\mathrm{from}\;(2):\;F_{B}=K_{B}\alpha_{B}$$

(20) $$F_{B}=K_{B}(\delta_{B}-\frac{U-\Omega L_{B}}{V_{B}})$$

The final stage is to put all the elements together.

(4) Compilation.

Using the assumed simplifications and substituting Relations (15) and (20) into Equations (7) and (10), respectively, one obtains:

(21) $$K_{A}(\delta_{A}(t)-\frac{U\left(t\right)+\Omega \left(t\right)L_{A}}{V})+K_{B}(\delta_{B}(t)-\frac{U\left(t\right)-\Omega \left(t\right)L_{B}}{V})=m(\dot{U}\left(t\right)+V\Omega \left(t\right))$$

(22) $$K_{A}(\delta_{A}(t)-\frac{U\left(t\right)+\Omega \left(t\right)L_{A}}{V})L_{A}-K_{B}(\delta_{B}(t)-\frac{U\left(t\right)-\Omega \left(t\right)L_{B}}{V})L_{B}=J\dot{\Omega }(t)$$

After making the appropriate calculations and simplifications, one obtains:

(23) $$m\dot{U}\left(t\right)+\frac{K_{A}+K_{B}}{V}U\left(t\right)+\frac{mV^{2}+K_{A}L_{A}-K_{B}L_{B}}{V}\Omega \left(t\right)=K_{A}\delta_{A}(t)+K_{B}\delta_{B}(t)$$

(24) $$J\dot{\Omega }(t)+\frac{K_{A}{L_{A}}^{2}+K_{B}{L_{B}}^{2}}{V}\Omega \left(t\right)+\frac{K_{A}L_{A}-K_{B}L_{B}}{V}U\left(t\right)=K_{A}L_{A}\delta_{A}(t)-K_{B}L_{B}\delta_{B}(t)$$

After performing a number of calculations, the form of the “bicycle model” for the local coordinate system was obtained. The appropriate further calculations need to be performed to obtain the form of the model for the global coordinate system.

Calculations against (X,Y) :

(25) $$V_{X}(t)=\dot{X}\left(t\right)=\sum F_{X}$$

(26) $$V_{Y}(t)=\dot{Y}\left(t\right)=\sum F_{Y}$$

(27) $$\dot{\psi }(t)=\Omega (t)$$

(28) $$\sum F_{X}=Vcos\psi -U(t)sin\psi \;(\mathrm{from}\;\mathrm{simplifications}\;\mathrm{adopted})$$

(29) $$\sum F_{Y}=Vsin\psi +U(t)cos\psi \;(\mathrm{from}\;\mathrm{simplifications}\;\mathrm{adopted})$$

(30) $$\dot{X}\left(t\right)=Vcos(\psi \left(t\right))-U(t)sin(\psi \left(t\right))$$

(31) $$\dot{Y}\left(t\right)=Vsin(\psi \left(t\right))+U(t)cos(\psi \left(t\right))$$

(32) $$X\left(t\right)=\int_{0}^{t}\dot{X}\left(\tau \right)d\tau =\int_{0}^{t}\left(V\cos\left(\psi \left(\tau \right)\right)-U\left(\tau \right)\sin\left(\psi \left(\tau \right)\right)\right)d\tau$$

(33) $$Y\left(t\right)=\int_{0}^{t}\dot{Y}\left(\tau \right)d\tau =\int_{0}^{t}(V\sin\left(\psi \left(\tau \right)\right)+U(\tau )\cos(\psi \left(\tau \right)))d\tau$$

(34) $$\psi \left(t\right)=\int_{0}^{t}\Omega \left(\tau \right)d\tau$$

Now the bicycle model is complete. Calculations for the local and global coordinate systems were made and presented.

Almost all authors dealing with the problems of vehicle motion control (both 2WS and 4WS) use the “bicycle model,” describing the “lateral dynamics” of the car in the form of two differential equations of the second order. Here, the “lateral dynamics” starter model is also the “bicycle model”.

## 4. Transmittance Formulation of the Model

**Figure 4:**
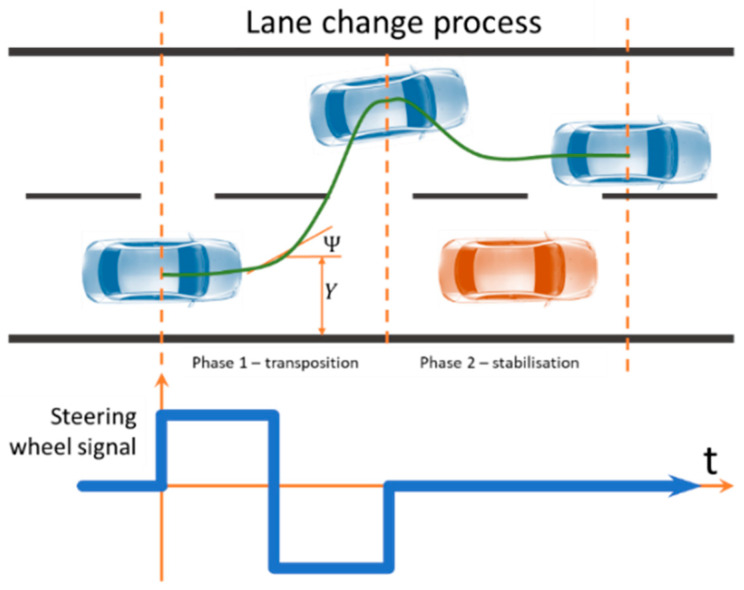
The concept of time decomposition of lane change control in a 2WS vehicle. Reprinted from Ref. [33].

The analysis of the 4WS bicycle model will be based on the research of the 4WS vehicle lane change process through a suddenly appearing obstacle. The driver or controller during this manoeuvre generates a “bang-bang” signal (Figure 4) [33]. According to the theory of optimal control, for time-optimal systems, meaning those systems that ensure that processes are realised in a minimum of time, control must take the form of “bang-bang” (infinite rapid ramp-up is possible when dealing with controllers that generate electronic signals). The “bang-bang” signal is a voltage signal generated by the controller. This signal is converted from electrical to mechanical by an actuator located in the vehicle. The actuator causes smoothing, delays, and takes the control signal to a more rounded structure. Between 0 and T, there is a jerk of the steering wheel to the left, a hold, and a jerk of the steering wheel to the right. Conversely, in the period from T to 2T, there is a hold, a jerk to the left again, and a return to the zero position. The period T is necessary to determine the duration of the obstacle avoidance process.

Figure 4 shows an example of the path taken by a vehicle during a lane change manoeuvre as a result of a suddenly appearing obstacle. It also finds reference in the results of simulation studies, but strictly, the trajectory of the vehicle during this manoeuvre depends on the speed at which it is travelling and the mechanical parameters of the vehicle.

In this model, we are dealing with linear equations in the local system and non-linear equations in the global system. In practise, the process of lane change takes place in such a way that the maximum values of the angle of vehicle deviation from the road axis are not high (only several degrees). This means that the non-linear variable transformation Equations (32) and (33) can be linearised. All equations for both systems are presented as follows:

- Linear equations of motion in the local coordinate system associated with the vehicle: Equations (23) and (24);
- Linear equations for the transformation of variables (as a result of linearization) from a local to a global system associated with a road: Equation (34) and

(35) $$X\left(t\right)=\int_{0}^{t}\dot{X}\left(\tau \right)d\tau =Vt$$

(36) $$Y\left(t\right)=\int_{0}^{t}\dot{Y}\left(\tau \right)d\tau =\int_{0}^{t}\left(V\psi \left(\tau \right)+U\left(\tau \right)\right)d\tau$$

After the equations are linearised, they can be subjected to the Laplace transform, thus obtaining the model in the form as follows:

(37) $$\left(ms+\frac{K_{A}+K_{B}}{V}\right)U\left(s\right)+\frac{mV^{2}+K_{A}L_{A}-K_{B}L_{B}}{V}\Omega \left(s\right)=K_{A}\delta_{A}\left(s\right)+K_{B}\delta_{B}\left(s\right)$$

(38) $$\left(Js+\frac{K_{A}{L_{A}}^{2}+K_{B}{L_{B}}^{2}}{V}\right)\Omega \left(s\right)+\frac{K_{A}L_{B}-K_{B}L_{B}}{V}U\left(s\right)=K_{A}L_{A}\delta_{A}\left(s\right)-K_{B}L_{B}\delta_{B}\left(s\right)$$

(39) $$Y\left(s\right)=\frac{V\psi \left(s\right)+U(s)}{s}$$

(40) $$\psi \left(s\right)=\frac{\Omega (s)}{s}$$

The equations will be presented in terms of the fundamental members of the dynamics. The next step is to change to the matrix form of Equations (37) and (38).

(41) $$\left[\begin{array}{c} U\left(s\right)\\ \Omega \left(s\right) \end{array}\right]={\left[\begin{array}{cc} ms+\frac{K_{A}+K_{B}}{V} & \frac{mV^{2}+K_{A}L_{A}-K_{B}L_{B}}{V}\\ \frac{K_{A}L_{A}-K_{B}L_{B}}{V} & Js+\frac{K_{A}{L_{A}}^{2}+K_{B}{L_{B}}^{2}}{V} \end{array}\right]}^{-1}\left[\begin{array}{cc} K_{A} & K_{B}\\ K_{A}L_{A} & {-K}_{B}L_{B} \end{array}\right]\left[\begin{array}{c} \delta_{A}\left(s\right)\\ \delta_{B}\left(s\right) \end{array}\right]$$

In order to derive the assumed values, it is now necessary to calculate the inverse matrix of the expression:

(42) $$A=\left[\begin{array}{cc} ms+\frac{K_{A}+K_{B}}{V} & \frac{mV^{2}+K_{A}L_{A}-K_{B}L_{B}}{V}\\ \frac{K_{A}L_{A}-K_{B}L_{B}}{V} & Js+\frac{K_{A}{L_{A}}^{2}+K_{B}{L_{B}}^{2}}{V} \end{array}\right]$$

To do this, count the determinant of the matrix, then the complement matrix, and finally transpose it. First, the determinant of matrix “*A*” will be counted.

(43) $$\det\left(A\right)=Jms^{2}+\frac{{m(K}_{A}{L_{A}}^{2}+K_{B}{L_{B}}^{2})+J(K_{A}+K_{B})}{V}s+\frac{K_{A}K_{B}{\left(L_{A}+L_{B}\right)}^{2}-mV^{2}(K_{A}L_{A}-K_{B}L_{B})}{V^{2}}$$

For easier analysis and to represent the individual parameters in terms of basic dynamics, the equation should be brought to a form where the last component in the equation is the number 1. To obtain this form of the equation, the last component of the expression should be taken out before the parenthesis. This will also help in obtaining such transmittance parameters as the time constant or the damping coefficient.

(44) $$\det\left(A\right)=\frac{K_{A}K_{B}{\left(L_{A}+L_{B}\right)}^{2}-mV^{2}\left(K_{A}L_{A}-K_{B}L_{B}\right)}{V^{2}}(T_{0}^{2}s^{2}+2\xi_{0}T_{0}s+1)$$

(45) $$A^{-1}=\left[\begin{array}{cc} \frac{Js+\frac{K_{A}{L_{A}}^{2}+K_{B}{L_{B}}^{2}}{V}}{det(A)} & \frac{-\frac{mV^{2}+K_{A}L_{A}-K_{B}L_{B}}{V}}{det(A)}\\ \frac{-\frac{K_{A}L_{A}-K_{B}L_{B}}{V}}{det(A)} & \frac{ms+\frac{K_{A}+K_{B}}{V}}{det(A)} \end{array}\right]$$

As a result, the matrix equation can be calculated. Due to the complex form of the determinant, the individual elements of the matrix were not counted immediately but replaced by parameters to facilitate the rest of the calculation.

(46) $$\left[\begin{array}{c} U\left(s\right)\\ \Omega \left(s\right) \end{array}\right]=\left[\begin{array}{cc} a_{11}(s) & a_{12}(s)\\ a_{21}(s) & a_{22}(s) \end{array}\right]\left[\begin{array}{cc} K_{A} & K_{B}\\ K_{A}L_{A} & {-K}_{B}L_{B} \end{array}\right]\left[\begin{array}{c} \delta_{A}\left(s\right)\\ \delta_{B}\left(s\right) \end{array}\right]$$

(47) $$U\left(s\right)=\left(a_{11}\left(s\right)K_{A}+a_{12}\left(s\right)K_{A}L_{A}\right)\delta_{A}\left(s\right)+(a_{11}\left(s\right)K_{B}-a_{12}\left(s\right)K_{B}L_{B})\delta_{B}\left(s\right)$$

(48) $$\Omega \left(s\right)=\left(a_{21}\left(s\right)K_{A}+a_{22}\left(s\right)K_{A}L_{A}\right)\delta_{A}\left(s\right)+(a_{21}\left(s\right)K_{B}-a_{22}\left(s\right)K_{B}L_{B})\delta_{B}\left(s\right)$$

(49) $$G_{U\delta_{A}}\left(s\right)=\frac{U(s)}{\delta_{A}\left(s\right)}=a_{11}\left(s\right)K_{A}+a_{12}\left(s\right)K_{A}L_{A},when\;\delta_{B}\left(s\right)=0$$

(50) $$G_{U\delta_{B}}\left(s\right)=\frac{U(s)}{\delta_{B}\left(s\right)}=a_{11}\left(s\right)K_{B}-a_{12}\left(s\right)K_{B}L_{B},when\;\delta_{A}\left(s\right)=0$$

(51) $$G_{\Omega \delta_{A}}\left(s\right)=\frac{\Omega (s)}{\delta_{A}\left(s\right)}=a_{21}\left(s\right)K_{A}+a_{22}\left(s\right)K_{A}L_{A},when\;\delta_{B}\left(s\right)=0$$

(52) $$G_{\Omega \delta_{B}}\left(s\right)=\frac{\Omega (s)}{\delta_{B}\left(s\right)}=a_{21}\left(s\right)K_{B}-a_{22}\left(s\right)K_{B}L_{B},when\;\delta_{A}\left(s\right)=0$$

**Figure 5:**
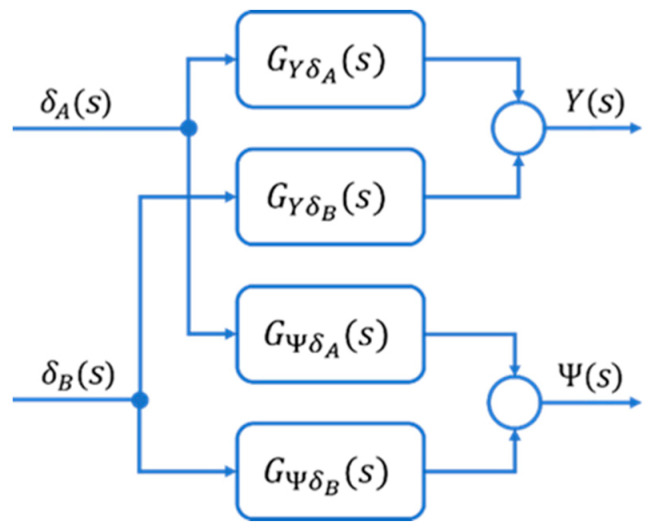
Model in the transfer functions version. Reprinted from Ref. [33].

Based on the calculations, the model was determined as a transfer function (Figure 5):

(53) $$G_{{Y\delta }_{A}}(s)=\frac{K_{Y\delta_{A}}\left(T_{Y\delta_{A}}^{2}s^{2}+2\xi_{Y\delta_{A}}T_{Y\delta_{A}}s+1\right)}{s^{2}(T_{0}^{2}s^{2}+2\xi_{0}T_{0}s+1)}$$

(54) $$G_{{Y\delta }_{B}}(s)=\frac{K_{Y\delta_{B}}(T_{Y\delta_{B}}^{2}s^{2}+2\xi_{Y\delta_{B}}T_{Y\delta_{B}}s-1)}{s^{2}(T_{0}^{2}s^{2}+2\xi_{0}T_{0}s+1)}$$

(55) $$G_{{\psi \delta }_{A}}(s)=\frac{K_{{\psi \delta }_{A}}{(T}_{\psi \delta_{A}}s+1)}{s(T_{0}^{2}s^{2}+2\xi_{0}T_{0}s+1)}$$

(56) $$G_{{\psi \delta }_{B}}(s)=\frac{K_{{\psi \delta }_{B}}\left(T_{\psi \delta_{B}}s+1\right)}{s\left(T_{0}^{2}s^{2}+2\xi_{0}T_{0}s+1\right)}$$

where:

(57) $$K_{Y\delta_{A}}=K_{Y\delta_{B}}=VK_{0}$$

(58) $$K_{{\psi \delta }_{A}}=K_{0}$$

(59) $$K_{{\psi \delta }_{B}}=-K_{0}$$

(60) $$K_{0}=\frac{K_{A}K_{B}\left(L_{A}+L_{B}\right)V}{K_{A}K_{B}{\left(L_{A}+L_{B}\right)}^{2}-mV^{2}\left(K_{A}L_{A}-K_{B}L_{B}\right)}$$

(61) $$T_{0}=V\sqrt{\frac{mJ}{K_{A}K_{B}{\left(L_{A}+L_{B}\right)}^{2}-mV^{2}\left(K_{A}L_{A}-K_{B}L_{B}\right)}}$$

(62) $$\xi_{0}=\frac{{m(K}_{A}{L_{A}}^{2}+K_{B}{L_{B}}^{2})+J(K_{A}+K_{B})}{2\sqrt{mJ(K_{A}K_{B}{\left(L_{A}+L_{B}\right)}^{2}-mV^{2}\left(K_{A}L_{A}-K_{B}L_{B}\right))}}$$

(63) $$T_{\psi \delta_{A}}=\frac{mL_{A}V}{K_{B}\left(L_{A}+L_{B}\right)}$$

(64) $$T_{\psi \delta_{B}}=\frac{mL_{B}V}{K_{A}\left(L_{A}+L_{B}\right)}$$

(65) $$T_{Y\delta_{A}}=\sqrt{\frac{J}{K_{B}\left(L_{A}+L_{B}\right)}}$$

(66) $$T_{Y\delta_{B}}=\sqrt{\frac{J}{K_{A}\left(L_{A}+L_{B}\right)}}$$

(67) $$\xi_{Y\delta_{A}}=\frac{L_{B}}{2V}\sqrt{\frac{K_{B}\left(L_{A}+L_{B}\right)}{J}}$$

(68) $$\xi_{Y\delta_{B}}=\frac{L_{A}}{2V}\sqrt{\frac{K_{A}\left(L_{A}+L_{B}\right)}{J}}$$

The above representation of the transfer functions shows a very strong connection between the transfer function parameters and the mechanical parameters. The transfer functions show the essence of the effects of steering angles on lateral and angular displacements [33].

**Figure 6:**
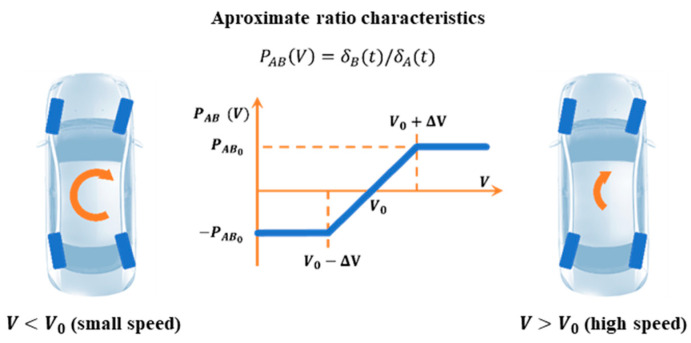
Overall idea of steering front and rear wheels in 4WS vehicles. Reprinted from Ref. [33]. Notation: *P_AB_*—gear ratio between front and rear wheel twist angles (*δ_A_*, *δ_B_*), *V*—vehicle speed, and *V*_0_—characteristic speed when *δ_B_* = 0.

In classic 4WS controls, the rear wheels are controlled by transforming the front wheel control accordingly (Figure 6) [33].

(69) $$P_{AB}\left(V\right)=\left\{\begin{array}{c} {{-P}_{AB}}_{0}\;for\;V<V_{0}-\Delta V\\ \frac{{P_{AB}}_{0}}{\Delta V}\left(V-V_{0}\right)\;for\;V_{0}\leq V\leq V_{0}+\Delta V\\ {P_{AB}}_{0}\;for\;V>V_{0}+\Delta V \end{array}\right.$$

**Figure 7:**
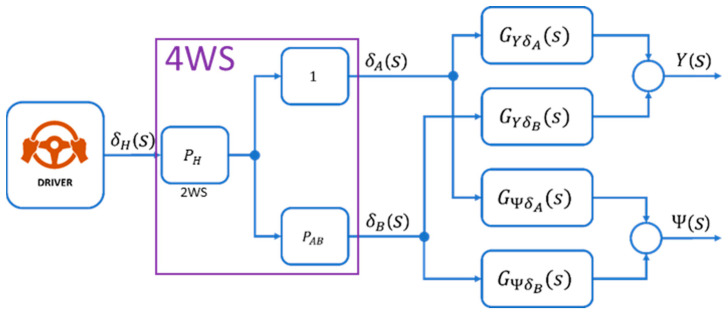
Model, including rear wheel ratio. Reprinted from Ref. [33].

With the assumed ratio characteristics, the transfer function model appears as follows (Figure 7) [33]:

(70) $$Y(s)=G_{Y\delta }(s)\delta (s)$$

(71) $$\mathrm{where}\;G_{Y\delta }(s)=G_{Y\delta_{A}}(s)+P_{AB}(V)G_{Y\delta_{B}}(s)$$

(72) $$\psi (s)=G_{\psi \delta }(s)\delta (s)$$

(73) $$\mathrm{where}\;G_{\psi \delta }\left(s\right)=G_{\psi \delta_{A}}\left(s\right)+P_{AB}\left(V\right)G_{\psi \delta_{B}}\left(s\right)$$

(74) $$\mathrm{from}:\;G_{Y\delta }\left(s\right)=\frac{K_{Y\delta }(T_{Y}^{2}s^{2}+2\xi_{Y\delta }T_{Y\delta }s+1)}{s^{2}\left(T_{0}^{2}s^{2}+2\xi_{0}T_{0}s+1\right)}$$

(75) $$G_{\psi \delta }\left(s\right)=\frac{{K_{\psi \delta }(T}_{\psi \delta }s+1)}{s\left(T_{0}^{2}s^{2}+2\xi_{0}T_{0}s+1\right)}$$

where:

(76) $$K_{Y\delta }=\left(1-P_{AB}\left(V\right)\right)VK_{0}$$

(77) $$T_{Y\delta }=\sqrt{\frac{J\left(\frac{1}{K_{B}}+\frac{P_{AB}\left(V\right)}{K_{A}}\right)}{\left(L_{A}+L_{B}\right)\left(1-P_{AB}\left(V\right)\right)}}$$

(78) $$\xi_{Y\delta }=\frac{L_{B}+P_{AB}\left(V\right)L_{A}}{2V\left(1-P_{AB}\left(V\right)\right)\sqrt{\frac{J\left(\frac{1}{K_{B}}+\frac{P_{AB}\left(V\right)}{K_{A}}\right)}{\left(L_{A}+L_{B}\right)\left(1-P_{AB}\left(V\right)\right)}}}$$

(79) $$K_{\psi \delta }=\left(1-P_{AB}\left(V\right)\right)K_{0}$$

(80) $$T_{\psi \delta }=\frac{mV\left(\frac{L_{A}}{K_{B}}-P_{AB}\left(V\right)\frac{L_{B}}{K_{A}}\right)}{\left(L_{A}+L_{B})(1-P_{AB}\left(V\right)\right)}$$

## 5. Concept of the Controller in the Steering System

**Figure 8:**
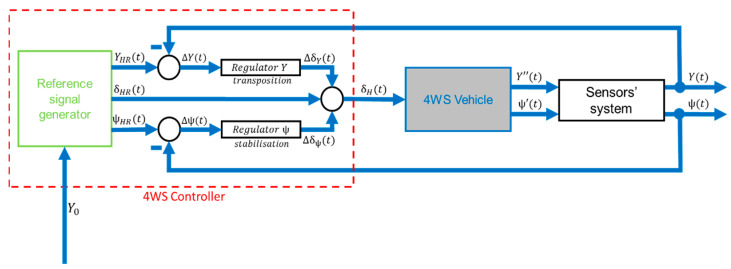
The 4WS control system.

The proposed detailed control system is shown below (Figure 8).

The proposed control system, through the reference signal generator, will generate three reference signals: $Y_{HR}(t)$ , $\psi_{HR}\left(t\right),$ and $\delta_{HR}(t)$ . Then they will be fed further into the system, except that in a feedback system, the subtraction of signals: $Y_{HR}(t)$ , $Y\left(t\right),$ $\psi_{HR}(t)$ , and ψ(t) will generate error signals: ΔY(t) and Δψ(t) . Hence, the need to select appropriate regulators for the correct operation of the system. Two regulators: Regulator Y and Regulator ψ will correct the signals ΔY(t) and Δψ(t) . The signals— $\Delta \delta_{Y}(t)$ and $\Delta \delta_{\psi }(t)$ are correction signals after the action of the regulators. The sum of the correction signals and the reference “bang-bang” signal $\delta_{HR}(t)$ will be the input signal for the vehicle ( δ(t) ). Regulators in this control system are based on the LQR method [36] (not mentioned in this article). 

**Figure 9:**
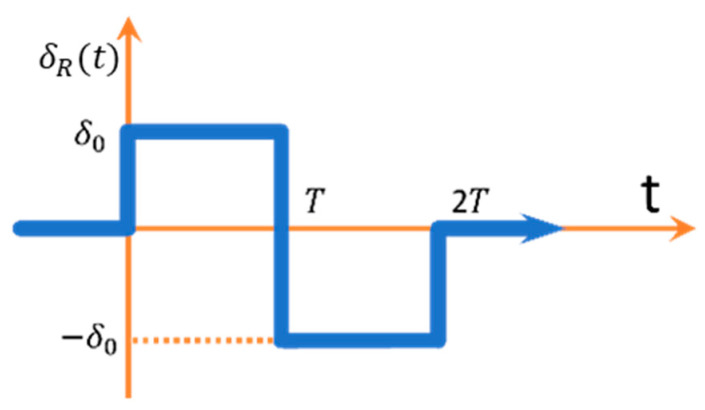
“Bang-bang” signal. Reprinted from Ref. [33].

The next step is to determine the form of the reference signal generator. For this, it is necessary to analyse the limits of the transfer functions. In particular, the “bang-bang” input waveforms (Figure 9) should be considered, as they approximate the real waveforms during the lane change process [33].

(81) $$\delta \left(t\right)=\delta_{0}(1\left(t\right)-2\cdot 1\left(t-T\right)+1(t-2T))$$

(82) $$\delta \left(s\right)=\delta_{0}\frac{{\left(1-e^{-sT}\right)}^{2}}{s}$$

(83) $$\delta_{A}\left(t\right)=\delta \left(t\right)$$

(84) $$when\;\delta_{B}\left(t\right)=P_{AB}(V)\delta \left(t\right)$$

(85) $$P_{AB}(V)>0$$

(86) $$\underset{t\to \infty }{\lim}\left(\psi \left(t\right)\right)=\underset{s\to 0}{\lim}\left(s\psi \left(s\right)\right)=\underset{s\to 0}{lim}\left(s(G_{\psi \delta_{A}}\left(s\right)\delta_{A}\left(s\right)+G_{\psi \delta_{B}}\left(s\right)\delta_{B}\left(s\right))\right)=0$$

(87) $$\underset{t\to \infty }{\lim}\left(Y\left(t\right)\right)=\underset{s\to 0}{\lim}\left(sY\left(s\right)\right)=\underset{s\to 0}{lim}\left(s\left(G_{Y\delta_{A}}\left(s\right)\delta_{A}\left(s\right)+G_{Y\delta_{B}}\left(s\right)\delta_{B}\left(s\right)\right)\right)=K_{Y\delta }\delta_{0}T^{2}$$

It was decided to use the model in a reduced version (members affecting only the transients are omitted), as this enables an analytical synthesis of the control of classical 4WS vehicles [33].

(88) $${G_{Y\delta }}_{R}(s)=\frac{K_{Y\delta }}{s^{2}}$$

(89) $${G_{\psi \delta }}_{R}(s)=\frac{K_{\psi \delta }}{s}$$

**Figure 10:**
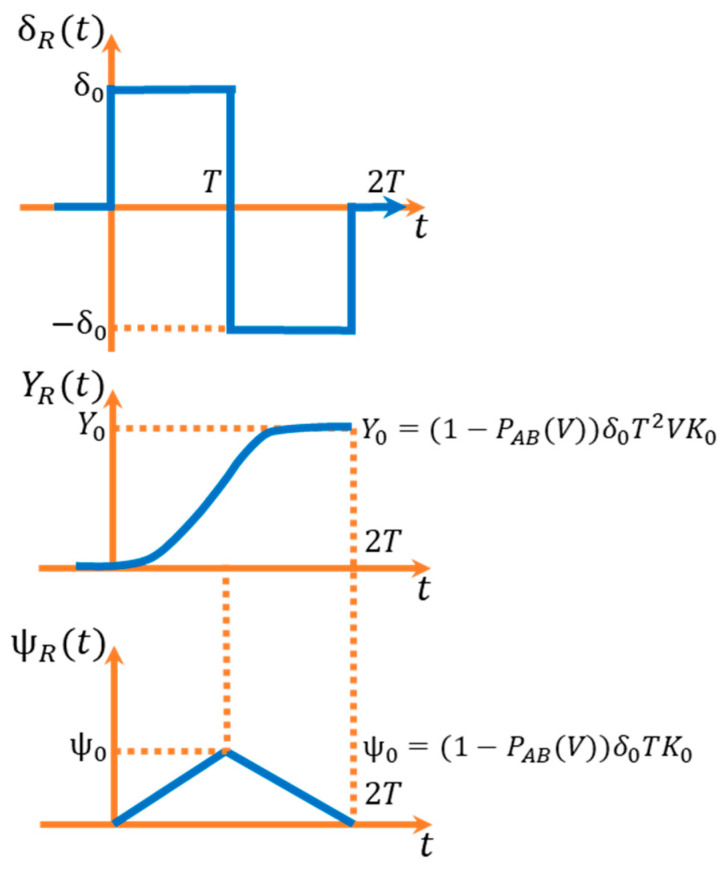
Reference signals in the control system. Reprinted from Ref. [33].

The same results of the boundary analysis are also obtained assuming a reduction of the initial transfer function model. The limit defined by Equation (87) is the steady-state displacement value $Y_{0}$ , as well as the magnitude of the lane change. It should be noted here that theoretically, according to Formula (86), the steady-state yaw angle is zero, so the vehicle moves on a track parallel to the original track. However, Equations (86) and (87) are not sufficient to determine the signal parameters $\delta_{0}$ and T . Therefore, an analogous study was performed here by updating the state after step excitation at time t=T, using (89). In this case, the angular displacement $\psi \left(T\right)=\psi_{0}$ reaches its maximum value $\psi_{0}$ defining the assumed linearity of the model. Based on these analyses, formulas for the values of T *i* $\delta_{0}$ can be calculated (Figure 10) [33]:

(90) $$T=\frac{Y_{0}}{V\psi_{0}}$$

(91) $$\delta_{0}=\frac{V\psi_{0}^{2}}{K_{\psi \delta }Y_{0}}$$

The form of the individual elements for the control process must be defined at the outset of the planned control concept. This mainly concerns the form of the reference signal generator and the vehicle, which will generate the real signals on the basis of the reference signals. In the feedback loop, so-called failures will arise as a result of the interaction of the vehicle’s individual parameters with the prevailing traffic situation [33].

**Figure 11:**
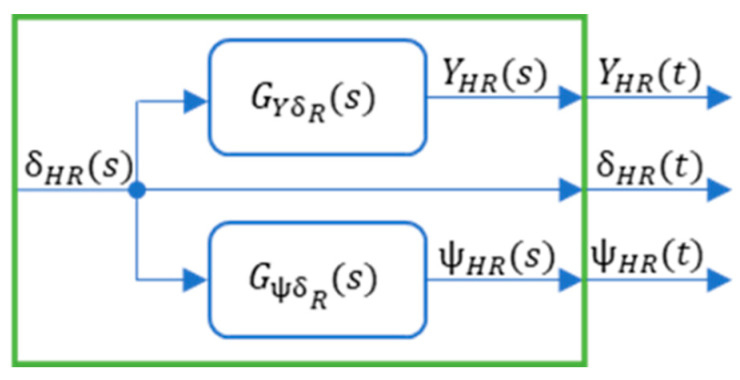
Reference signal generator.

The reference signal generator will include simplified transfer functions (Figure 11).

(92) $$\delta_{HR}(s)=p\cdot \delta_{R}(s)$$

where p is the steering gear ratio, and the signals $Y_{HR}(s)$ and $\psi_{HR}(s)$ are the effects of the signal $\delta_{HR}(s)$ on the reduced transmittance forms. 

## 6. Concept of the Measurement System

**Figure 12:**
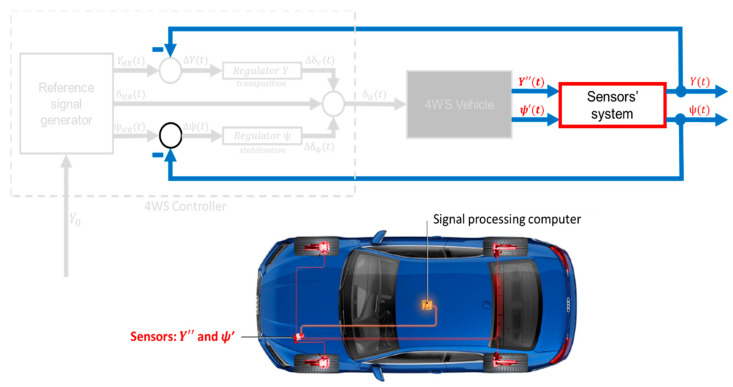
Measuring system.

The model of the measuring system must represent how the measuring system obtains information from the vehicle regarding its actual movements. It must provide these to the controller in order to minimise the deviation signals by adjusting between the reference and actual signals, which translates into correcting the vehicle’s path so that the actual vehicle’s path is as close as possible to the reference (Figure 12). This is put in descriptive terms.

The measuring system has the task of continuously providing the controller with information about two signals—the lateral displacement Y(t) and the angular displacement ψ(t) . For this purpose, sensors can be used in the ESP (Electronic Stability Program), sometimes referred to as ESC (Electronic Stability Control), which is obligatory in every car. For its operation, it measures, among other things, the lateral acceleration of the vehicle’s centre of mass $\ddot{Y}\left(t\right)$ and the yaw angular velocity $\dot{\psi }(t)$ . At this point, to obtain the desired displacement values, the transverse acceleration signal must be integrated twice and the yaw angular velocity signal must be integrated once.

The use of existing sensors on the vehicle makes it much easier to identify the parameters of the control signal, as it does not require the development of additional circuitry and coupling to the existing one.

## 7. Concept of the 4WS Virtual Vehicle

**Figure 13:**
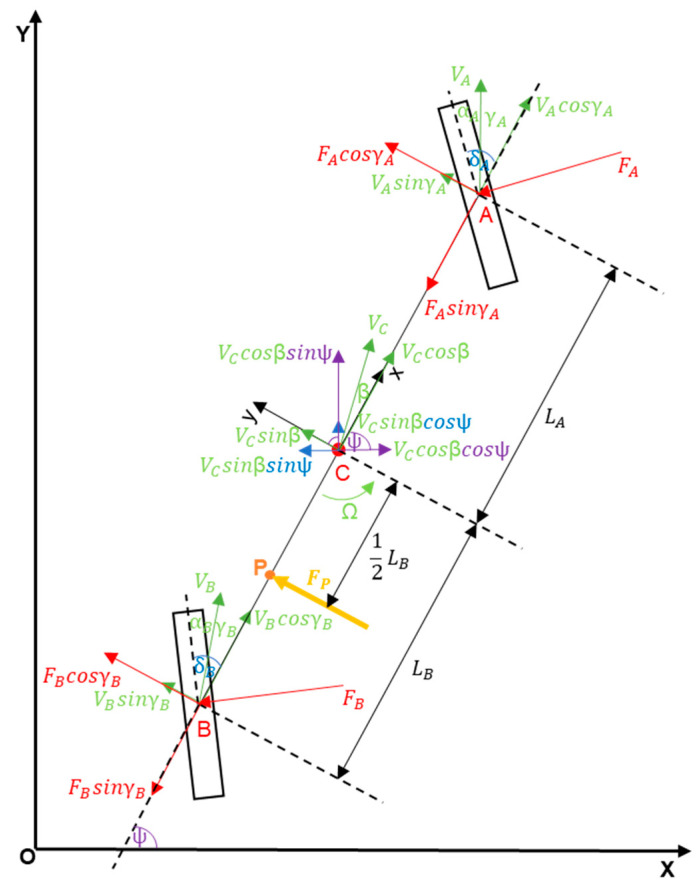
Including the action of crosswind force in the bicycle model.

Since the authors do not have an actual 4WS vehicle, there is a need to create a virtual facility. It is assumed that the vehicle, when changing lanes, travels at a constant speed on a level road without excessive skidding and braking in front of an obstacle but avoids it immediately. As a result of this assumption, the influence of the braking and suspension systems can be ignored in the construction of the virtual model due to the small nature of the excursions. In addition, during lane changes, the vehicle’s yaw angles are small (on the order of a few degrees), making the vehicle’s side-to-side tilt of little importance, so this element can also be ignored. On the other hand, the steering system plays an important role when vehicle deflection is small; hence, the 4WS virtual vehicle model consists of a model of the steering system and a model of vehicle movement. The model of vehicle movement is described by a non-linearized 4WS bicycle model with the effect of crosswind forces (as a result of the assumptions described earlier)—Figure 13 [36].

The lateral force acts on the car body at a point called the centre of pressure (*P*), which generally does not coincide with the vehicle’s centre of mass. Therefore, when passing through a crosswind area, a yaw moment acting on the car is created, which can lead to a change of direction [37]. Assuming that the vehicle is turning left during a lane change, point *P* should be placed closer to the rear axle of the vehicle. For ease of calculation, the location of the centre of pressure is assumed to be at a distance of 0.5 $L_{B}$ from the centre of mass. The effect of the force $F_{P}$ is described as follows:

(93) $$F_{p}=\frac{A_{y}}{A_{y_{0}}}A\frac{\rho_{p}V_{r}^{2}}{2}c_{F_{y}}$$

where:

(94) $$V_{r}^{2}={\left(V+V_{w}\cos\psi \right)}^{2}+V_{w}^{2}{sin}^{2}\psi$$

(95) $$c_{F_{y}}=2.48\beta^{0.382}$$

(96) $$c_{M_{z}}=2\beta^{1.77}$$

(97) $$\beta =arctg\left(\frac{V_{w}\sin\psi }{V+V_{w}\cos\psi }\right)$$

where: $A_{y}$ —lateral area, $A_{y_{0}}$ —the lateral surface of the car body directly exposed to wind, A —front surface, $\rho_{p}$ —air density, $V_{w}$ —wind speed, $c_{F_{y}}$ —dimensionless lateral force coefficient, and $c_{M_{z}}$ —dimensionless yaw moment coefficient.

The calculation of the moment of force $F_{p}$ takes into account the change from the dimensionless lateral force coefficient $c_{F_{y}}$ to the dimensionless yaw moment coefficient $c_{M_{z}}$ . The value of $V_{w}$ should be taken as 20 m/s ± 3 m/s [37]. The modified equations in the local system are shown below (model of vehicle movement system):

(98) $$m\dot{U}\left(t\right)+\frac{K_{A}+K_{B}}{V}U\left(t\right)+\frac{mV^{2}+K_{A}L_{A}-K_{B}L_{B}}{V}\Omega \left(t\right)=K_{A}\delta_{A}\left(t\right)+K_{B}\delta_{B}\left(t\right)+F_{P}$$

(99) $$J\dot{\Omega }\left(t\right)+\frac{K_{A}{L_{A}}^{2}+K_{B}{L_{B}}^{2}}{V}\Omega \left(t\right)+\frac{K_{A}L_{A}-K_{B}L_{B}}{V}U\left(t\right)=K_{A}L_{A}\delta_{A}\left(t\right)-K_{B}L_{B}\delta_{B}\left(t\right)-\frac{F_{p}L_{B}}{2}$$

**Figure 14:**
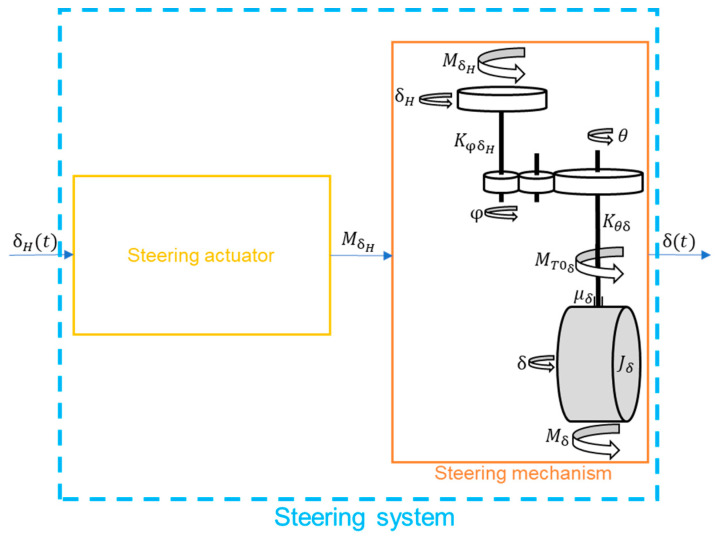
Steering system of the 4WS virtual vehicle.

The model of the steering system is presented below (Figure 14) [38]:

(100) $$\frac{K_{s_{UK}}}{T_{s_{UK}}^{2}s^{2}+2\xi_{s_{UK}}T_{s_{UK}}s+1}\;(\mathrm{steering}\;\mathrm{actuator}—\mathrm{second-order}\;\mathrm{inertial}\;\mathrm{member})$$

(101) $$J_{\delta }\ddot{\delta }\left(t\right)=-\mu_{\delta }\dot{\delta }\left(t\right)+M_{\delta }\left(t\right)+\frac{{{(K}_{\varphi \delta }}_{H}+K_{\theta \delta }){(\delta }_{H}\left(t\right)-p\delta \left(t\right))}{p{K_{\varphi \delta }}_{H}+K_{\theta \delta }}\;(\mathrm{steering}\;\mathrm{mechanism})$$

where $J_{\delta }$ —moment of inertia of the steering knuckle with wheel, $\mu_{\delta }$ —damping factor in the stub axle bearing, $M_{\delta }$ —moment of external force acting on the steering knuckles, $M_{T0\delta }$ —moment of friction of the dry bearing of the steering knuckle, ${K_{\varphi \delta }}_{H}$ —stiffness coefficient of the steering shaft, $K_{\theta \delta }$ —stiffness coefficient of the shaft representing the rod, p —gear ratio, δ —angle of rotation of the road wheel knuckle, θ —angle of rotation of the gear wheels from the knuckles, φ —angle of rotation of the gear wheel from the steering wheel side, and $\delta_{H}$ —handlebar rotation angle.

**Figure 15:**
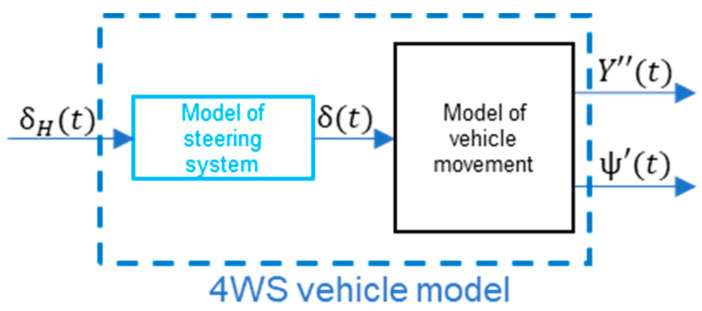
The 4WS vehicle model.

The full model of the 4WS virtual vehicle is presented below (Figure 15):

## 8. Preliminary Simulations

The research is carried out entirely in a simulation environment due to the lack of a real 4WS car facility. Only the parameters of the “lateral dynamics” model necessary for the operation of the model controller and the model of vehicle movement of the virtual vehicle were subjected to the identification process (not mentioned here). The identifications were carried out on the real 2WS vehicle because the authors did not have a 4WS vehicle. Note that both the parameter set of the “lateral dynamics” model and the technique for identifying these parameters are the same for 2WS and 4WS vehicles.

Simulation studies make it possible to investigate certain issues that are impossible to investigate in a real facility. In terms of sensitivity studies, for example, it is possible to address issues of the effect of an increase in steering system on the developed algorithm, which cannot be determined in a real vehicle in the short term. In addition, simulations make it possible to theoretically test the proposed control method without having a real object.

As a result of the unavailability of a real vehicle, simulation studies were carried out based on records of data, partly from the literature [38] and partly from our own research (not mentioned here). The necessary data for the simulation are shown in the following table (Table 1).

**Figure 16:**
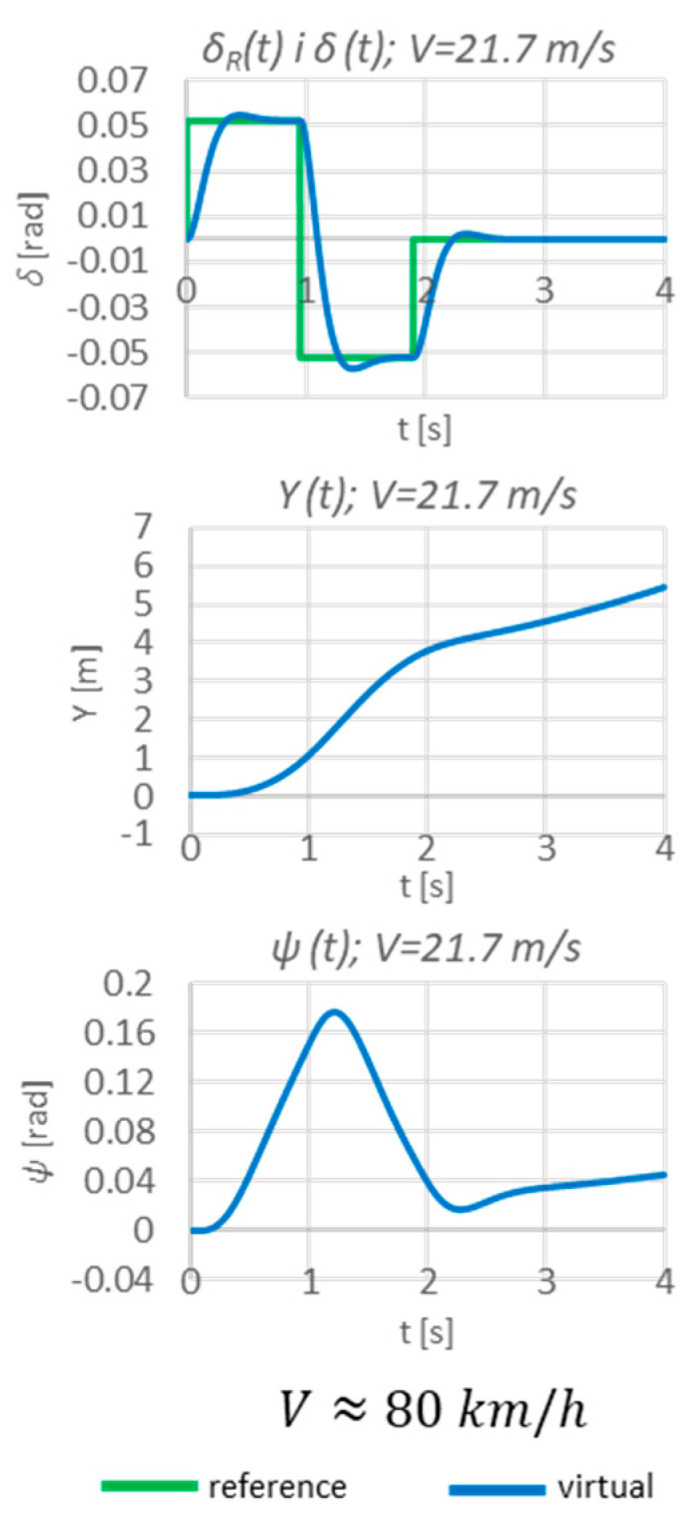
Simulation results in an open-loop system.

At first, the test was carried out in an open-loop (without feedback) system at one speed (21.7 m/s ≈ 80 km/h) in order to confirm the necessity of the proposed system (Figure 16). 

**Figure 17:**
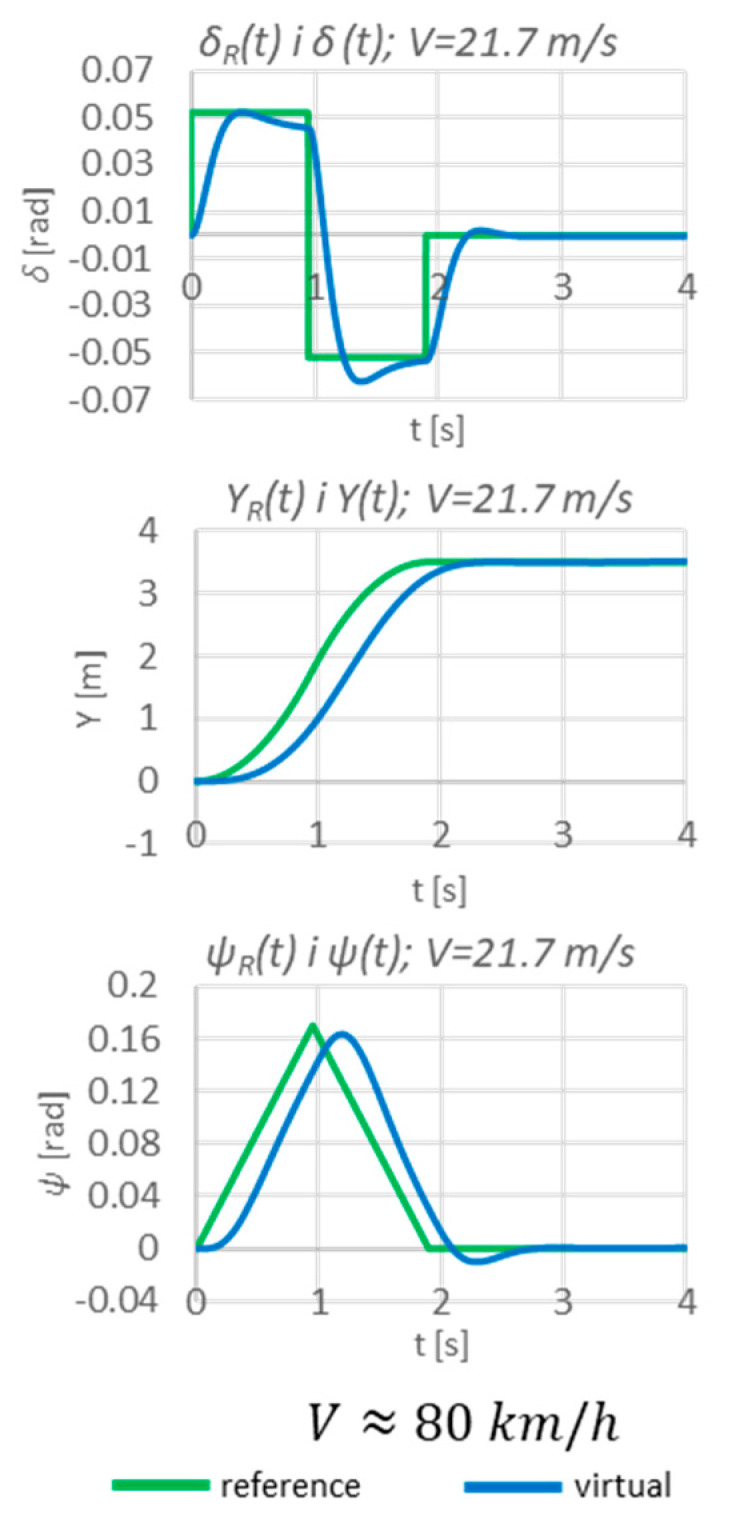
Simulation results in a closed-loop system.

In an open-loop system, the vehicle is unable to achieve the assumed steady-state values, hence the need for a closed-loop system controller (as proposed so far; Figure 17).

In a closed-loop system, the vehicle achieves the intended displacement.

The essence of the authors’ idea is a lane change controller based on a fairly simple mathematical model. Simulation tests are to check the sensitivity of its operation, taking into account implementation imperfections. An important part of this research (presented in the article) concerns the impact of measurement imperfections (noise, offset) on the effects of lane change control.

The quality of closed-loop control is measured on the basis of lapse-based control quality indicators (four indicators were used). However, due to the volume of the work and the presentation of test results on the example of only one speed, these indicators were not presented.

The experiment consists of checking the correct functioning of the developed algorithm and determining whether it is sensitive to possible sensor disturbances.

## 9. Sensitivity Testing Due to Disturbances in Sensors

Interference occurs when the desired information is read out by the measurement system, which then processes the acquired data accordingly and delivers it to the controller. A distinction is made between interference due to noise and offset. Noise distorts the signal read by the measuring system, and the offset makes the location of the start of the read-out signal out of line with its actual state. As in previous studies, the individual components affecting the distortion will be considered separately, but for several values of both noise and offset.

**Figure 18:**
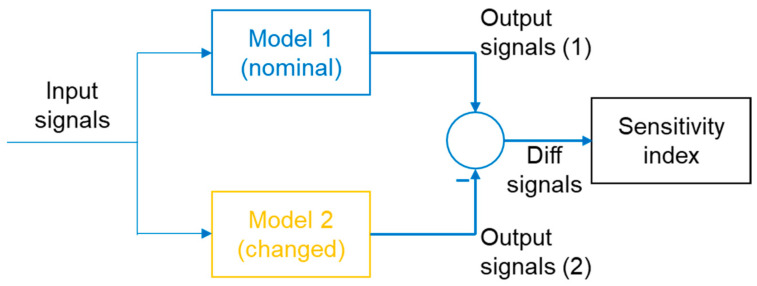
Sensitivity analysis scheme. Reprinted from Ref. [5].

An example numerical index (sensitivity index) was used to assess the measure of system sensitivity. A schematic of the system sensitivity analysis is shown in Figure 18.

An example of the sensitivity index used is shown below [5]:

(102) $$W_{x}=100\frac{\int_{0}^{\tau }{\left(x_{1}\left(t\right)-x_{2}\left(t\right)\right)}^{2}}{\int_{0}^{\tau }{\left(x_{1}\left(t\right)\right)}^{2}}$$

**Figure 19:**
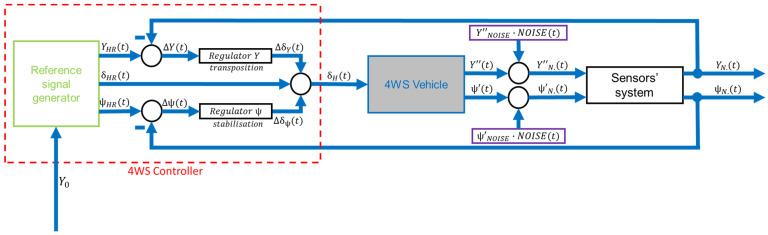
Control system, including noise.

Sensitivity studies will begin by checking the effect of noise on the performance of the algorithm. A modified schematic of the control system incorporating noise into the system is shown below (Figure 19).

The noise was added to the signals measured by the sensors’ systems. In this solution, the sensors’ system measures values such as transverse acceleration and angular velocity. It then integrates them accordingly to obtain the values of the individual displacements. Additionally, as a result of the noise, the transverse acceleration and angular velocity are integrated with it. White noise was used in the sensitivity study, as it is the standard type of noise used when studying the effect of noise on the developed closed-loop automatic control system. Furthermore, it is possible for it to occur in the signals measured by the sensors’ systems, and it is available in the Simulink software library.

The form of the noise introduced is as follows [5]:

(103) $${Y^{\prime \prime }}_{N.}\left(t\right)=Y^{\prime \prime }\left(t\right)+{Y^{\prime \prime }}_{NOISE}\cdot NOISE\left(t\right)$$

(104) $${\psi^{\prime }}_{N.}\left(t\right)=\psi^{\prime }\left(t\right)+{\psi^{\prime }}_{NOISE}\cdot NOISE\left(t\right)$$

where:

$NOISE\left(t\right)$ —standard white noise signal (amplitude = 1),
${Y^{\prime \prime }}_{NOISE}$ , ${\psi^{\prime }}_{NOISE}$ —amplitude of the noise signal.

Table 2 shows the values of noise parameters that could really occur in the analysed system.

**Figure 20:**
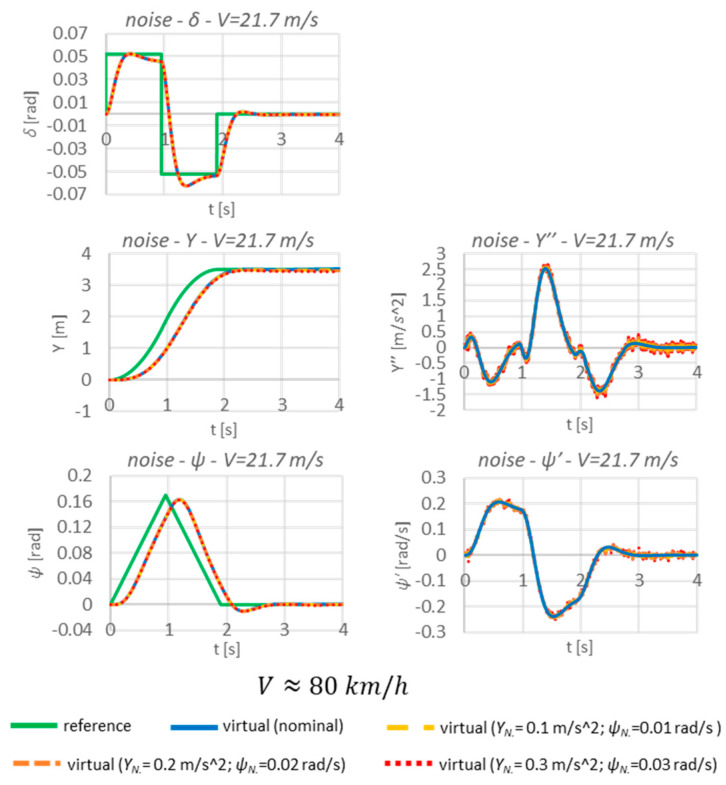
Sensitivity tests due to the presence of noise.

The simulations with noise are shown below (Figure 20).

The presented sensitivity tests of the algorithm show that the noise occurring does not disrupt the functioning of the whole system. The differences between the nominal and disturbed signals are very small—there is virtually no difference. This is evidenced by the almost identical system responses and the very low values of the sensitivity indexes (Table 3).

In the case of the system proposed by the authors, the presence of noise in the measured signals does not greatly affect the final form of the displacements. However, in order to obtain information on the correctness of the algorithm’s development, this element should always be checked.

**Figure 21:**
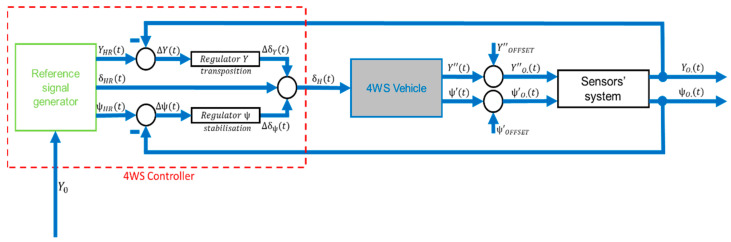
Control system, including offset.

The next element in the sensitivity tests will be to check the performance of the algorithm when an offset occurs (Figure 21). The scope of these tests follows the same pattern as for the presence of noise.

The form of the offset introduced is as follows [5]:

(105) $${Y^{\prime \prime }}_{O.}\left(t\right)=Y^{\prime \prime }\left(t\right)+{Y^{\prime \prime }}_{OFFSET}$$

(106) $${\psi^{\prime }}_{O.}\left(t\right)=\psi^{\prime }\left(t\right)+{\psi^{\prime }}_{OFFSET}$$

where:

${Y^{\prime \prime }}_{OFFSET}$ , ${\psi^{\prime }}_{OFFSET}$ —constant value.

Table 4 shows the values of offset parameters that could really occur in the analysed system.

**Figure 22:**
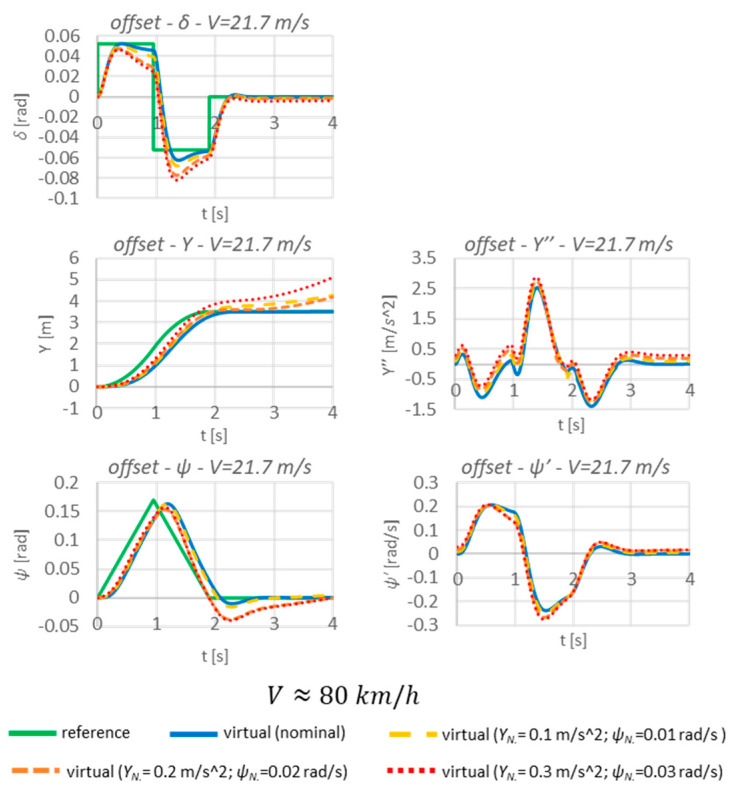
Sensitivity tests due to the presence of offset.

The simulations with offset are shown below (Figure 22).

The below results clearly show that the developed algorithm is quite sensitive to the occurrence of offsets. Even at the smallest set values of this disturbance, the controller is unable to adjust and stabilise the vehicle to achieve the desired displacements. This translates into an increase in the values of the sensitivity indexes (Table 5).

It can be seen that the greatest disparity between the nominal responses and the responses taking into account the offset occurs in the final phase. Hence, the values of the sensitivity indices, despite the unfavourable trajectory of the run, are not high. Nevertheless, among the conclusions of the tests carried out, the use of some sort of filtering element should be considered to prevent the occurrence of offsets. Otherwise, the system will not be able to function properly. However, this issue will remain to be improved in future research.

## 10. Conclusions

This study shows how important and significant a role the “bicycle model” plays in the overall concept of 4WS vehicle control. Its role, however, could not be highlighted without embedding it in a suitable environment, taking into account, in addition to the research part discussed, the vehicle’s sensor system. The systems that provide its path and tracking information to the controller are fundamental to the ability of the whole system to function, as without the feedback enabled by the sensors, it would not be possible to provide the vehicle with real-time (online) information about changes occurring in and around the vehicle. 

The preliminary studies carried out clearly demonstrate the validity of a system with feedback. On the other hand, based on sensitivity tests, it can be concluded that with certain disturbances, the proposed algorithm is able to function correctly (noise), but there are also those that preclude its correct functioning (offset). This leads to the conclusion that this element should be focused on in order to improve the functioning of the algorithm.

The approach presented here allows the operation of the algorithm to be demonstrated in a closed-loop automatic control system. The form of the model in the controller is much simpler compared to the virtual vehicle model. The simulations carried out show that the developed algorithm works correctly. In addition, the extremely simplified model requires a small number of parameters, which, if these parameters need to be updated in real-time (online), should not be a problem to implement in a short time.

In future work, it is envisaged to develop research on improving the functioning of the developed algorithm and to extend the issue of sensitivity testing to other elements. 

## Figures and Tables

**Table 1:** Vehicle’s data.

Name	Parameter	Unit	Value
velocity	V	$\left[m/s\right]$	21.7
mass	m	[kg]	1627
mass moment of inertia	J	$\left[kg\cdot m^{2}\right]$	2893
distance between A and C	$L_{A}$	[m]	1.15
distance between B and C	$L_{B}$	[m]	1.56
yaw coefficients to p. A	$K_{A}$	$\left[N/rad\right]$	57,719
yaw coefficients to p. B	$K_{B}$	$\left[N/rad\right]$	80,723
characteristic velocity	$V_{0}$	$\left[m/s\right]$	15
increase in velocity	ΔV	$\left[m/s\right]$	5
gear ratio between wheels	${P_{AB}}_{0}$	[-]	0.1
set value	$Y_{0}$	[m]	3.5
peak value	$\psi_{0}$	[rad]	0.17
moment of inertia	$J_{\delta }$	$\left[kg\cdot m^{2}\right]$	0.2
damping factor	$\mu_{\delta }$	$\left[Nms/rad\right]$	100
moment of external force	$M_{\delta }$	[Nm]	0
moment of friction	$M_{T0\delta }$	[Nm]	0
stiffness coefficient	${K_{\varphi \delta }}_{H}$	$\left[Nm/rad\right]$	100
stiffness coefficient	$K_{\theta \delta }$	$\left[Nm/rad\right]$	1 × 10^11^
gain factor	$K_{s_{UK}}$	[-]	1
time constant	$T_{s_{UK}}$	[-]	0.1
damping factor	$\xi_{s_{UK}}$	[-]	0.7
gear ratio	p	[-]	16.4
wind speed	$V_{w}$	$\left[m/s\right]$	20

**Table 2:** Parameters of noise [5].

Parameter	Unit	Value
${Y^{\prime \prime }}_{NOISE}$	$\left[m/s^{2}\right]$	0.1; 0.2; 0.3
${\psi^{\prime }}_{NOISE}$	[rad/s]	0.01; 0.02; 0.03

**Table 3:** Sensitivity indexes for noise.

$W_{\delta }$ [%]	$W_{Y}$ [%]	$W_{\psi }$ [%]
1.1 × 10^−7^	2.8 × 10^−3^	1.7 × 10^−3^
4.5 × 10^−7^	0.01	6.9 × 10^−3^
1.1 × 10^−6^	0.03	0.02

**Table 4:** Parameters of offset [5].

Parameter	Unit	Value
${Y^{\prime \prime }}_{OFFSET}$	$\left[m/s^{2}\right]$	0.1; 0.2; 0.3
${\psi^{\prime }}_{OFFSET}$	[rad/s]	0.01; 0.02; 0.03

**Table 5:** Sensitivity indexes for offset.

$W_{\delta }$ [%]	$W_{Y}$ [%]	$W_{\psi }$ [%]
0.82	1.38	0.3
5.47	0.78	6.27
9.76	6.1	6.91

## Data Availability

Data is contained within the article.
